# A Mobility Enabled Inpatient Monitoring System Using a ZigBee Medical Sensor Network

**DOI:** 10.3390/s140202397

**Published:** 2014-01-30

**Authors:** Hoi Ching Tung, Kim Fung Tsang, Ka Lun Lam, Hoi Yan Tung, Benjamin Yee Shing Li, Lam Fat Yeung, King Tim Ko, Wing Hong Lau, Veselin Rakocevic

**Affiliations:** 1 Department of Electronic Engineering, City University of Hong Kong, Tat Chee Avenue, Kowloon, Hong Kong, China; E-Mails: cherry.tung@cityu.edu.hk (H.C.T.); kalunlam@cityu.edu.hk (K.L.L.); hytung@cityu.edu.hk (H.Y.T.); yeesli2-c@my.cityu.edu.hk (B.Y.S.L.); eelyeung@cityu.edu.hk (L.F.Y.); eektko@cityu.edu.hk (K.T.K.); itwhlau@cityu.edu.hk (W.H.L.); 2 School of Engineering & Mathematical Science, City University London, Northampton Square, London EC1V 0HB, UK; E-Mail: V.Rakocevic@city.ac.uk

**Keywords:** ZigBee medical sensor network, mobility management, patient monitoring

## Abstract

This paper presents a ZigBee In-Patient Monitoring system embedded with a new ZigBee mobility management solution. The system enables ZigBee device mobility in a fixed ZigBee network. The usage, the architecture and the mobility framework are discussed in details in the paper. The evaluation shows that the new algorithm offers a good efficiency, resulting in a low management cost. In addition, the system can save lives by providing a panic button and can be used as a location tracking service. A case study focused on the Princes of Wales Hospital in Hong Kong is presented and findings are given. This investigation reveals that the developed mobile solutions offer promising value-added services for many potential ZigBee applications.

## 1. Introduction

Aging is a problem that knows no boundaries. The worldwide population over age 65 is expected to more than double from 357 million in 1990 to 761 million by 2025. In most of the developed regions such as Western Europe, or Japan, elderly people already account for one-fifth of the total population and the ratio of workers to retirees will drop to 2:1. Needless to say, aging will decrease business productivity and gradually affect national economies. In addition, longevity also implies high medical expenditure for different age-related disabilities and diseases, such as Alzheimer's [1].

Following the recent maturation of the information and communication technologies, telemedicine has become a well-recognized solution for outpatient monitoring solutions to reduce medical costs as well as the workload of medical practitioners. Telemedicine has been one of the hottest research topics in recent years. Hii gave a general idea of how to achieve health monitoring using smart phonea [2] and Custodio and his co-workers provided a review of different potential communication technologies for healthcare applications [3]. On the other hand, Chen and his team have proposed an intelligent knowledge-based home care system for watchdog purposes [4]. Within a general framework of telehealth and telemedicine, some authors devoted have their efforts to medical sensor design. A visual sensor has been presented to detect the abnormal activity of an outpatient [5] and a portable bio-potential acquisition system with flexible PDMS dry electrodes was presented in [6] which captured ECG signal anytime and from anywhere. Further to this, the design of a fall detection system has been discussed in [7]. Security and protocol design are also active areas for telemedicine research. A strong authentication protocol design for healthcare applications was put forth by Kumar and his colleagues [8], while a wireless medical sensor network protocol supporting dynamic resource allocation was explored by Sousa [9]. Although these previous works have been done by different research teams, Wireless Sensor Networks (WSNs) are recognized as the essential part of all solutions.

A WSN generally serves as the front end data collection medium. It links up all medical sensors and relays the medical data to the central platform. ZigBee, WiFi and Bluetooth 4.0 are the most popular WSNs. Reliability of connection accounts for the topmost important priority of ZigBee. WiFi and Bluetooth 4.0 use a star and star bus topology as the network architecture, respectively. The system breaks down if the master node fails. However, ZigBee deploys a mesh capability which will not suffer from a single point of failure. With its reliable network structure, ZigBee has been widely adopted by different homecare systems [10–17]. ZigBee—as a “three-low” solution: low cost, low power consumption and low data rate—presents a promising solution for patient monitoring inside hospitals. In addition, ZigBee supports 128-bit Advanced Encryption Standard (AES) encryption to provide strong security. Generally, it is a network layer protocol based on the IEEE 802.15.4 standard [18–19] and it is an ideal candidate for wireless personal area network (WPAN) communications. ZigBee originated from embedded applications requiring low power consumption and low data rates [10–22].

Although ZigBee has been proved to be suitable for data transmission inside operation rooms and intensive care units [17], there is a lack of complete design for ZigBee inpatient monitoring. In this paper, a novel ZigBee In-Patient Monitoring system (ZIPMS), supporting patient tracking, patient monitoring as well as personal paging, is developed. ZIPMS not only automatically collects medical readings from patients, but also simultaneously tracks the location of patients. By simply pressing a panic button, medical assistance can be invoked immediately and medical practitioners may easily figure out the exact location of the patient in a very short time to provide timely assistance. With slight modification, ZIPMS can further facilitate the operation of outpatient clinics and also helps to page patients to return to the hospital for medical treatment or examination.

It has been well documented that the non-mobile design of ZigBee [18,23–24] leads to invalid medical reading collection for the mobile sensors [25]. To enable ZigBee mobility, ZIPMS sensors and routers have equipped a newly defined ZigBee Mobility Manager (ZiM2) which provides handoff management, paging and location management. The detailed design of ZiM2 will be described in a later section.

To showcase the applicability of ZIPMS, a network planning case study, based on the Main Clinical Block and Trauma Center of the Prince of Wales Hospital in Hong Kong, is presented in this paper. The Prince of Wales Hospital was founded about 30 years ago and it was the first hospital in the Eastern New Territories of Hong Kong. More importantly, the Prince of Wales Hospital is a major public and teaching hospital which is affiliated with the Medical Faculty of the Chinese University of Hong Kong. The Prince of Wales Hospital was chosen as the case study because it carries all the common characteristics of major hospitals in Hong Kong.

The remaining sections of this paper are organized as follows: the system overview of ZIPMS will be described in Section 2. The design of ZiM2 will be discussed in Section 3 and the address assignment will be discussed in Section 4. A network planning case study in the Prince of Wales Hospital and the performance evaluation of ZIPMS will be given in Sections 5 and 6, respectively. Furthermore, the social impact of the presented technology will be discussed in Section 7 and a conclusion will be drawn in Section 8.

## 2. ZigBee Inpatient Monitoring System Overview

As discussed in Section 1, the ZIPMS system is proposed as a solution to alleviate the aging problem. The use of the ZIMPS system lowers the cost of medical service and also reduces the workload of medical specialists. More importantly, ZIPMS assures that patient will receive first aid in the golden time (6 min after injury). According to Red Cross statistics, the survival rates of individuals suffering cardiac arrest decreases by approximately 7 to 10% every minute that defibrillation is delayed. Therefore, time is a very critical factor. ZIPMS is a total solution to resolve the captioned emergency in hospitals since it includes location tracking, panic button and medical data monitoring. When the ZIPMS receives a panic alert or medical warning alert, the system will locate the patient and broadcast the emergency signal to associated medical specialists with the patient's location and information. Under such a circumstance, the medical specialists can provide the assistance as soon as possible.

Before the system design and architecture is studied, the services of ZIPMS are explained. The services are:

1. Medical parameter monitoring
2. Patient location tracking
3. Patient paging

The medical parameter monitoring service collects medical data from patients regularly. It saves the data to the database and allows the doctor to retrieve the data at a later time. If the collected data indicates a danger level, the system will inform the doctor instantly. The medical parameters that are monitored by ZIPMS are listed in Table 1.

**Figure 1.**
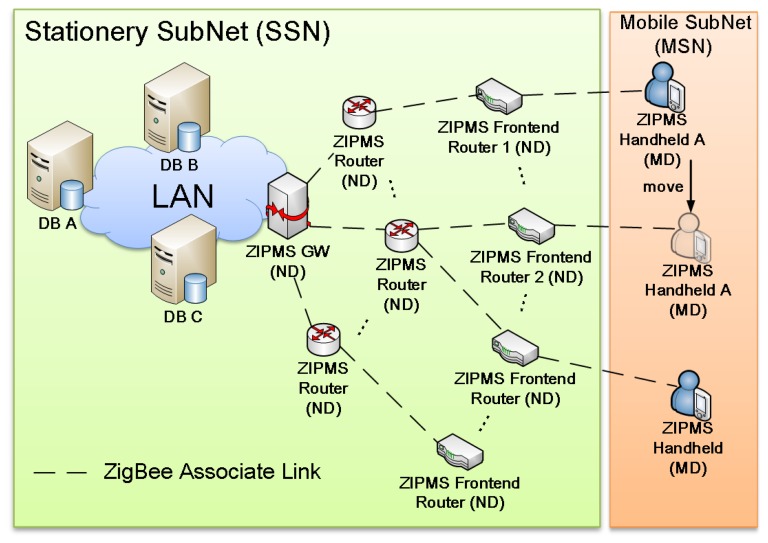
The ZigBee in-patient monitoring system (ZIPMS) overview.

ZIPMS actively performs auto-diagnosis to determine the conditions of patients after collecting the medical data. As a result, the alert signal can be issued by the patients via the panic button or ZIPMS. After receiving the alert signal, ZIPMS identifies the location of the corresponding patients. Finally, the medical specialist can provide the assistance instantly. Of course, a similar function can also be applied to out-patients. More importantly, the ZIPMS can act as a queuing management system. The control centre of the hospital can easily trace the location of each patient. In addition, it will make decision on the next patient to be examined by doctors. The ZIPMS will page the patient 15 min before the doctor will inspect him. The ZIPMS overview is shown in Figure 1.

Since most hospitals have already implemented Local Area Networks (LANs), ZIPMS will complement the LAN. In general, ZIPMS is responsible for the frontend data transmission while the LAN takes care the backbone transmission. Consequently, the installation cost is reduced. In Figure 1, database servers (DBs) A, B and C are the centralized database servers connecting with LAN that host all the patient information and they update each other periodically and automatically. For security purposes, they are scattered in different locations and the role of the primary DB can be switched between them on an as need basis. In addition, based on the LAN structure, the workload can be shared amongst different DBs. Therefore the operation rate can be speeded up and single point failure will be avoided, and thus a higher throughput can be obtained.

In Figure 1, there are two parts in the ZIPMS, namely the Stationery Subnet (SSN) and the Mobile Subnet (MSN). The reason of preferring ZigBee for SSN to WiFi is that ZigBee possesses a mesh capability and is scalable. ZigBee is a fully mesh network that would not suffer from a single point failure and the devices (stationary) can be relocated easily due to wireless nature.

The SSN consists of Networking Devices (ND) such as ZIPMS gateway, ZIPMS routers and ZIPMS frontend routers which route data from the source to destination. Typically, the ZIPMS gateway is embedded with a ZigBee coordinator and translates the ZigBee signal to the Ethernet signal in order to serve as an intersection point of the LAN and the ZigBee medical sensor network. Both the ZIPMS routers and the ZIPMS frontend routers are equipped with ZigBee routers and they perform the typical tasks as normal routers. The only difference between the ZIPMS routers and the ZIPMS frontend routers is that the ZIPMS frontend routers support ZiM2 while the ZIPMS router does not. On the other hand, MSN is formed by Medical Devices (MDs) which are ZigBee end devices. MDs are generally referred as ZIPMS handhelds which integrates sensors and panic buttons. Patients may carry MDs around the hospital and forward the user requests and medical data to SSN.

Based on [19], the NDs are designed as Full Function Devices (FFDs) which are equipped with routing capability and they can communicate with both FFD and Reduced Function Device (RFD). In ZIPMS, direct communications between MDs are not required, therefore MDs are designed as RFD to lower the implementation cost as well as enjoy longer battery life time.

## 3. The Proposed ZigBee Mobility Manager

In contrast to the former ZigBee healthcare system, ZIPMS enables ZigBee mobility in order to provide simultaneous patient monitoring service. ZigBee was originated for non-mobile application, thus the current standard does not cater for mobility management capability. In this paper, a mobile management profile, namely ZigBee Mobility Manager (ZiM2), has been defined to enable the mobility of ZIPMS. ZiM2 is designed to provide location management, handoff management and paging which operate with ZIPMS frontend routers and ZIPMS handhelds. Based on the described need, ZiM2 is designed in two parts: mobile and frontier to operate with handheld and frontend routers, respectively. ZiM2 mobile performs the handoff procedure while ZiM2 frontier works with the location databases to provide location management and paging services.

In the ZIPMS, ZiM2 is designed to satisfy the QoS requirement. The handoff strategy is regarded as the most challenging part in the design of ZigBee mobility solution. A good handoff strategy ensures ZIPMS handheld a good QoS. However, existing solutions [23–24] are relatively fragile in providing QoS since they are basically non-mobile systems. In these solutions, a mobile ZigBee device will change its parent only when the communication with the original parent has been lost. During the handover period, the service of ZigBee mobile device is suspended. Thus typical solutions do not guarantee end to end QoS. The ZIPMS is equipped with ZiM2, hence service suspension will not appear since ZiM2 keeps the communication of the incoming and outgoing parents for a short while, thus provoking seamless transition. Under such circumstance, the end to end QoS of the ZIMPS handheld will not be deteriorated by the handoff. The details of ZiM2 handheld profile will be presented in the next section.

### 3.1. ZiM2 Mobile

Basically, the handoff decision is based on the signal strength which normally is measured by Received Signal Strength Indicator (RSSI). In essence, the Link Quality Indicator (LQI) is given instead of the RSSI in characterizing the signal strength of a ZigBee device. In order to conform to ZigBee specifications [19], LQI will be used thereafter.

**Figure 2.**
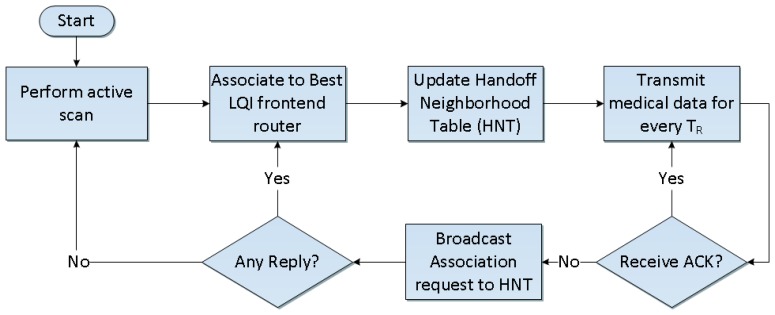
ZiM2 mobile.

Summarizing from the previous section, the ZiM2 mobile is designed for ZIPMS handheld devices to handle the handoff procedure from ZIPMS frontend router 1 to ZIPMS frontend router 2. The detailed handoff procedure is shown in Figure 2.

Under normal circumstances, the ZIPMS handheld will perform an active scan to locate ZIPMS when it is powered on. After completing association to the best LQI ZIPMS frontend router, the ZIPMS handheld will update the Handoff Neighborhood Table which stores the short addresses of nearby frontend routers. Referring to Figures 1 and 2, the ZIPMS handheld is associated to the ZIPMS frontend router 1 originally. ZIPMS handheld sends out the medical data every T_R_ seconds. As long as the ZIPMS handheld moves toward ZIPMS frontend router 2, the ZIPMS handheld will not receive the acknowledgment from ZIPMS frontend router 1 anymore. Therefore, the ZIPMS handheld invokes a fast handoff procedure. With HNT, the ZIPMS handheld can directly broadcast association requests to the frontend routers (listed in HNT) such as ZIPMS frontend router 2 without *performing* scanning. After receiving the replies from the HNT frontend routers, the handheld will then attach to the device with the best LQI. Comparing to the standard orphan scan procedure, less time is used and so the data loss probability will also be reduced. If no reply is received from HNT frontend routers, the ZIPMS handheld will perform an active scan again. The active scan is initialized here instead of an orphan scan because an orphan scan only realigns the orphan device with its original parent (referred as ZIPMS frontend router 1 in this example). Since a ZIPMS handheld is a mobile device which needs to be attached to a new parent, active scan is applied. ZiM2 and WiFi use similar handoff approach, they first disassociate from original parent/access point. Then, they scan the environment and hunt for a new parent/access point to associate. Attention is drawn to the fact that ZiM2 has incorporated HNT to reduce the frequency of performance scanning which reduces the energy consumption and accelerate the handoff process.

### 3.2. ZiM2 Frontier

**Figure 3.**
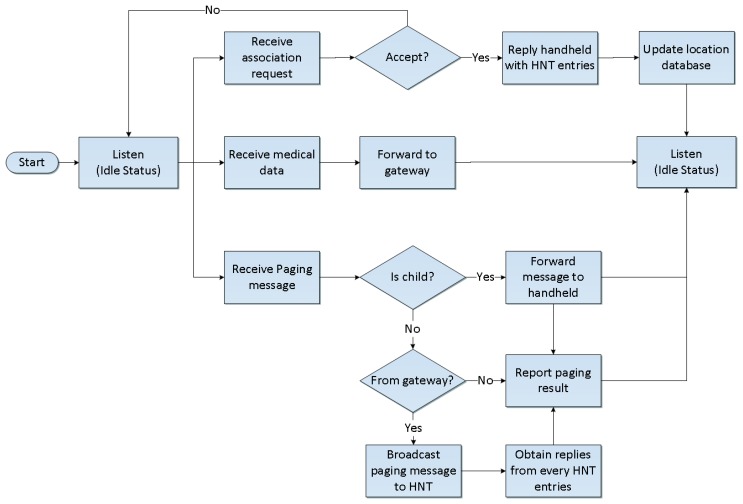
ZiM2 frontier.

The ZiM2 frontier is designed for ZIPMS frontend routers and works in harmony with the location database to provide location updating and paging services. The location databases reside in distributed locations of a hospital as shown in Figure 1 carry the location information of all ZIPMS handhelds. ZiM2 frontier will update the location information when a ZIPMS frontend router accepts association request from ZIPMS handheld. If ZIPMS pages a specific handheld, its location information must be obtained from the database beforehand. The detailed operation of ZiM2 frontier is shown in Figure 3.

During the daily operation, the ZIPMS frontend router may receive three types of signals such as association requests, medical data and paging messages. In Figure 3, the location update procedure is initiated by a receiving association request. After accepting such a request from the handheld, the frontend router extracts the data entries of other frontend routers from its neighborhood table and sends the data to the handheld. When the association has been completed, the frontend router will update the location database. For example, the frontend router 2 changes the current location of handheld A from the short address of frontend router 1 to its short address. Apart from the location update, the ZIPMS frontend router forwards the collected medical data to the gateway in order to set up the personal medical profile for every patient.

Paging is another challenge for frontier ZiM2 because improper design of the paging mechanism leads to broadcast flooding. In order to avoid flooding, the paging region must be minimized. However, the coverage of ZigBee is relatively small and so ZIPMS handheld performs handoffs frequently to maintain its network connection. Under this situation, a handheld may already leave before the frontend router receives paging message. To solve this dilemma, ZiM2 frontier forwards the paging message to its frontend router neighbor. Therefore, the handheld can be located even when the paging region is kept to a minimum. The developed ZiM2 fully considers the QoS requirement for the mobile applications. Important features of the ZiM2 include:

- Fast handoff ability, the probability of message loss due to handoff is minimized.
- Instant location update to capture the location of handheld.
- Optimal paging region to solve the dilemma between frequency handoff and broadcast flooding

Such an approach can minimize the probability of service suspension due to handoffs.

## 4. ZIPMS Network Address Assignment

Network address assignment is an important issue for ZigBee network because it determines both the routing mechanism and the network capacity. The current standard of ZigBee supports two address assignment mechanisms: tree and stochastic. Tree address assignment scheme is applied to the ZIPMS because it provides a quicker response comparing to stochastic approach and this is quite important in handling the mobile ZIPMS handheld. Furthermore, tree address assignment scheme provides the tree structure network which enables the tree route capability which has been proved to be reliable under a hospital environment [26]. Subsequently, the number of route requests is reduced, and hence the broadcast flooding is minimized.

In the ZIPMS, network addresses are assigned using a distributed addressing scheme to provide a finite sub-block of network addresses to NDs such as ZIPMS gateway, ZIPMS router and frontend router. These addresses are unique within a particular network and are assigned by a ND to its children (e.g., other NDs or ZIPMS handhelds). The ZIPMS gateway has the authority to determine the capacity of the network and the maximum number of children allowed within a network. Amongst these children, a maximum of nwkMaxRouters are assigned as router-capable devices while the remaining routers are reserved as ZIPMS handhelds. Every device is associated with a depth number which indicates the minimum number of hops in the path from the device to the ZIPMS gateway. The ZIPMS gateway itself has a depth of zero, while its children have a depth of one. Multi-hop ZIPMS networks will have a maximum depth number of less than one. The ZIPMS gateway also determines the maximum depth of the network. Define Cskip(d) as the size of the address sub-block being distributed by each ND at that depth to its router-capable child devices for a given network depth d, hence:

(1) $$\text{Cskip}(d)=\{\begin{array}{cc} 1+Cm\;\cdot \;(Lm-d-1), & Rm=1\\ \frac{1+Cm-Rm-Cm\;\cdot \;Rm^{Lm-d-1}}{1-Rm}, & Rm\neq 1 \end{array}$$

where nwkMaxChildren (Cm) = the maximum number of children under a ND; nwkMaxDepth (Lm) = the maximum depth in the network; nwkMaxRouters (Rm) = the maximum number of ZIPMS router and frontend router under a ND.

If a device has Cskip(d) = 0, it does not accept children and will be treated as a ZIPMS handheld in this research. A ND having Cskip(d) > 0 accepts child devices and assigns unique addresses to them depending on whether the child device is router-capable or not. Network addresses are assigned to router-capable child devices with the value of Cskip(d) as an offset. A ND assigns an address, which is greater than its own address by unity, to its first router-capable child device. Subsequently assigned addresses to router-capable child devices are separated from each other by Cskip(d). A maximum value of nwkMaxRouters of such addresses can be assigned. Network addresses shall be assigned to ZIPMS handheld in a sequential manner with the nth address, A_n_, given by the following equation:

(2) $$A_{n}=A_{\text{pareant}}+\text{Cskip}(d)\cdot Rm+n$$

where 1 ≤ n ≤ (Cm − Rm) and A_ND_ represents the address of ND. An address sub-block cannot be shared between two devices, it is possible that one parent exhausts its list of addresses while a second parent has unused addresses. A ND having no available address does not permit a new device to join the network. In this situation, the new device is compelled to look for another MD. If no other NDs are available within the transmission range of the ZIPMS handheld, the device will be unable to join the ZIPMS network unless it is physically moved.

## 5. ZIPMS Network Planning

A network planning case study of ZIPMS for the Main Clinical Block and Trauma Center of Prince of Wales Hospital in Hong Kong is conducted using the information in [24,27,28]. This section discusses how to construct a ZigBee tree network for the captioned studied site in order to illustrate ZIPMS deployment in a realistic situation. To gain more insight, an experiment is set up to evaluate the performance of ZIPMS in the next section.

**Figure 4.**
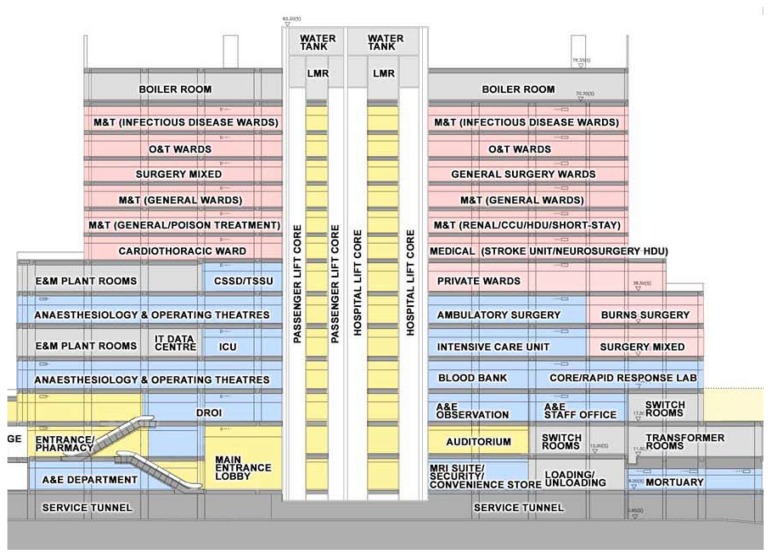
Schematic of the main clinical block and trauma center.

Prior to going into the detailed steps, a brief introduction of the studied site is given. The Prince of Wales Hospital was built in the 1970s. To alleviate the pressure of increasing population, the Main Clinical Block and Trauma Center was built in 2006 and started to serve citizens in 2010. The details of the Center and typical ward settings are tabulated in Tables 2 and 3, respectively, while the schematic of the center is shown in Figure 4.

With better understanding of testing site, the attention is drawn to the network planning for ZIPMS. After performing an onsite connectivity test, it was found that the communication radius was 35 m and the ZigBee signals could penetrate two floors. For example, the ZigBee device on the 3rd floor could directly communicate with ZigBee device on 1st floor using one single hop.

To construct a ZigBee tree network for ZIPMS, the value of Rm, Cm and Lm must be determined. In ZIPMS, Rm refers to total number of ZIPMS routers and ZIPMS frontend routers and Cm refers to the total number of devices including handhelds, routers and frontend routers that a ND can hold. The value of Lm relates to the signal penetration ability and geographic of the working site.

**Figure 5.**
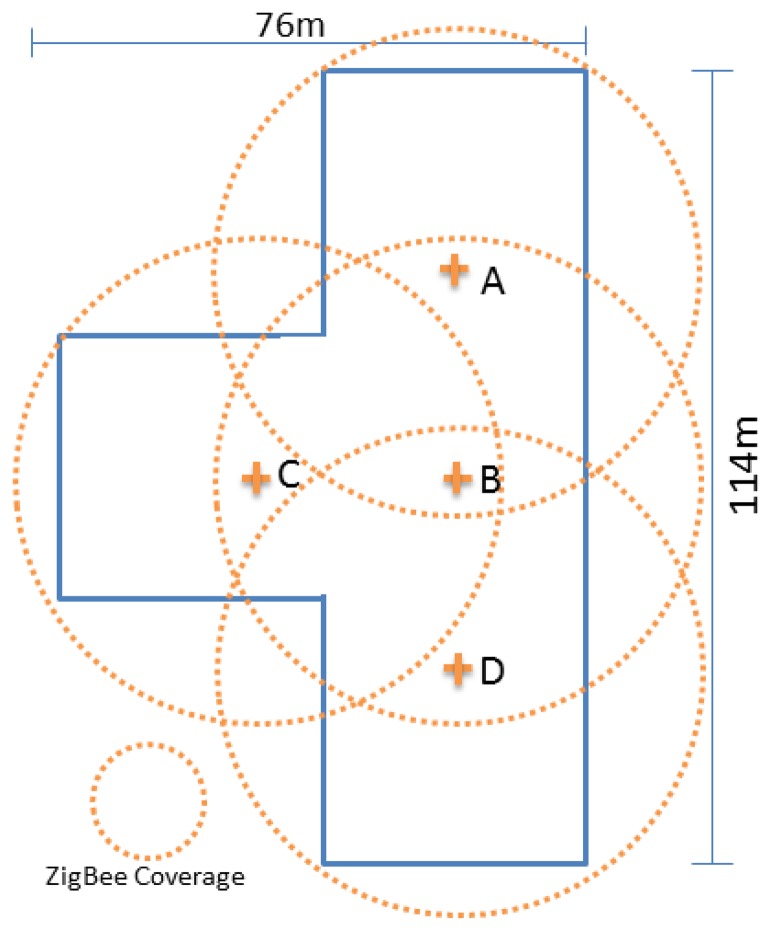
Simplified floor plan.

In the simplified floor plan of the Trauma center (Figure 5), four ZIPMS frontend routers are needed for every floor and only one frontend router (indicating as B in Figure 5) is sufficient to connect to all the other three frontend routers. As a result, the frontend router B serves as a parent of the other three frontend routers and a three layer sub-tree network including ZIPMS handheld has been set up.

**Figure 6.**
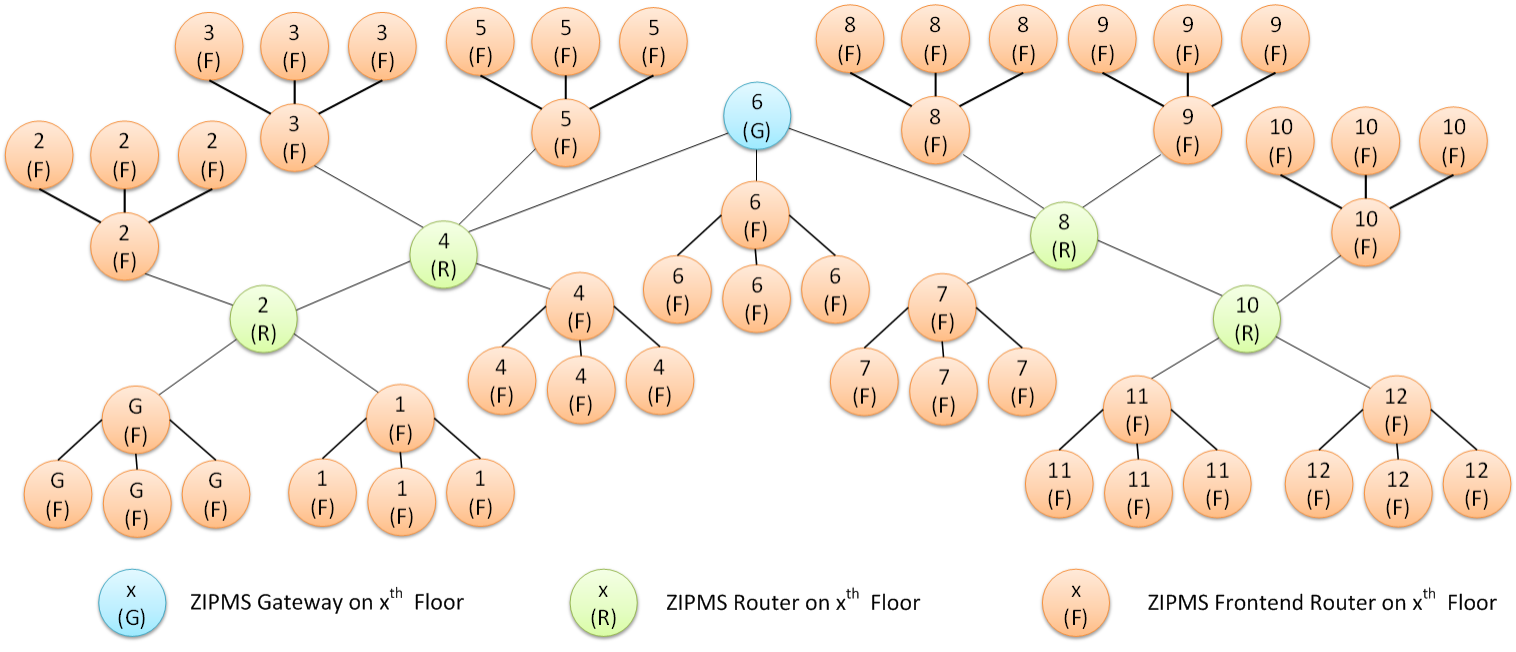
ZIPMS tree network.

The next issue is to figure out the minimum number of ZIPMS routers that connect the ZIPMS frontend routers to the ZIPMS gateway. The transmission delay is reduced by minimizing Lm, thus the ZIPMS gateway is located on 6th floor. As mentioned in earlier text, ZigBee signal can penetrate two floors, so ZIPMS routers are inserted on 2nd, 4th, 8th and 10th floors in order to propagate the ZigBee signals. Finally, a ZIPMS tree network (Figure 6) has been obtained.

Figure 6 illustrates the ZIPMS tree network without the ZIPMS handheld. Therefore, the tree network is six-layer, i.e., Lm = 6. Apart from Lm, Rm can be found out from Figure 6. Since both ZIPMS router 6 and ZIPMS router 10 host four other NDs which is the maximum number among the entire network. Hence, the Rm of ZIPMS 4, i.e., Rm = 4. After obtaining Rm and Lm, Cm for ZIPMS can be calculated by using Equation (3):

(3) $$\text{CSkip}(\text{d})\cdot \text{Rm}\leq 2^{16}$$

where d = 0; i.e., after calculation, Cm is 48 and the network configurations of ZIPMS for the Main Clinical Block and Trauma Center of Prince of Wales Hospital are summarized as Table 4.

From Tables 2 and 3, there are three wards on each floor and 40 beds for each ward and this implies 40 ZIPMS handhelds and 120 handhelds are required for each ward and floor, respectively. A single ZIPMS frontend router can host 48 handhelds, implying one ZIPMS frontend router is sufficient for a ward. Since there are four ZIPMS frontend routers on each floor and in principle, 192 handhelds can be accommodated (i.e., 60% more than the requirements, doubling the capacity). Based on the analysis, it is concluded that the network configuration proposed here can fulfill the daily operation requirement of the studied center.

## 6. Performance Evaluation of ZIPMS

**Figure 7.**
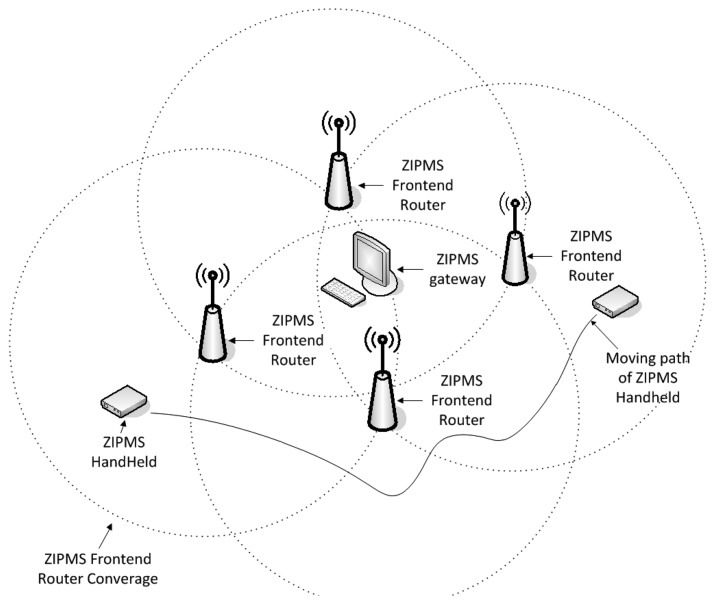
ZIPMS.

**Figure 8.**
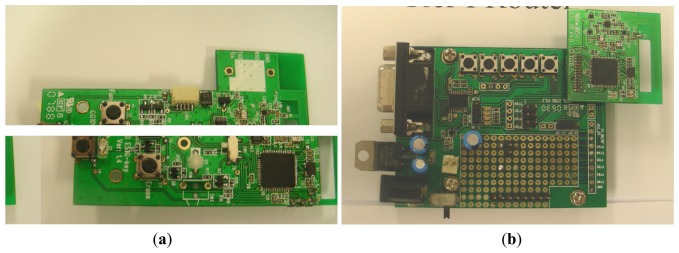
ZIPMS: (**a**) handheld (**b**) frontend router/gateway.

**Figure 9.**
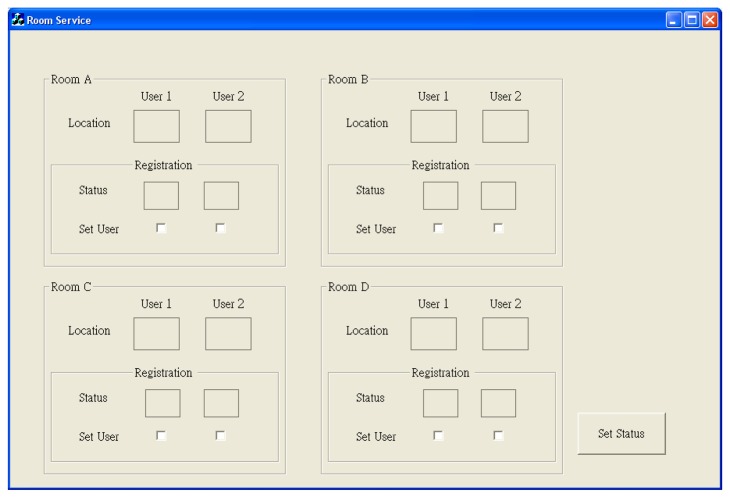
User interface of the ZIPMS prototype.

Since ZiM2 has enabled the mobility of ZIPMS, it is important to evaluate ZIPMS under a mobile environment. In addition, reliability is another significant issue for medical application. Therefore, the performance of ZIPMS is evaluated with co-existing WiFi. To carry out the experiment, a prototype (Figures 7, 8 and 9) for ZIPMS is developed and the details will be given in the later text.

The ZigBee system of ZIPMS consists of the block diagram (Figure 7), prototype gateway and devices (Figure 8) and the User Interface (Figure 9). In Figure 7, numerous ZIPMS frontend routers are anchored into proper positions to provide good signal strength wireless connectivity. The ZIPMS handhelds are then well connected to the gateway virtually at any point within the network. In the experiment, there were two handhelds (Figure 8a), four frontend routers (Figure 8b) and a gateway (Figure 8b). *Z*IPMS features location tracking of patients so that rescue action may be provided in case of emergency.

In Figure 9, the User Interface (UI) shows the current location of each handheld. When a handheld attaches to a new frontend router, the UI updates immediately to show its latest position. A panic button was incorporated into the handheld. When the user presses the panic button, the UI will indicate the location with red color and the name of device will be shown immediately. Subsequently, medical practitioners may be sent to the spot and an acknowledgement reply signal will be sent to the panic handheld. The bi-directional communication between the handheld and the gateway was also used in the patient paging service.

**Figure 10.**
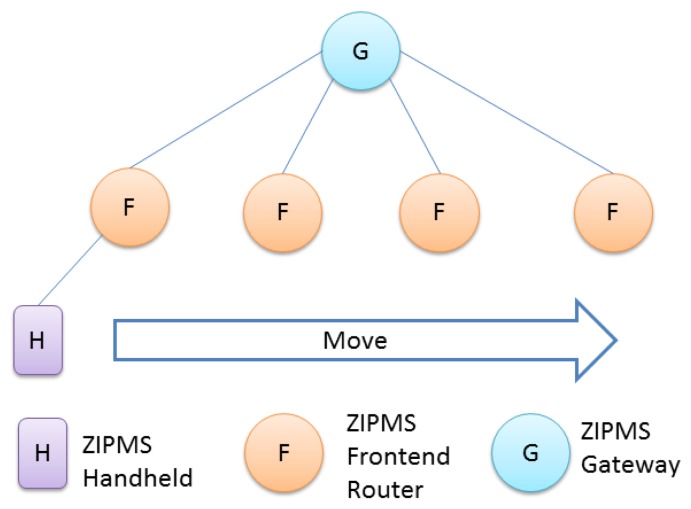
Experimental network.

The prototype was also used to setup an experiment to evaluate the reliability of ZIPMS. Another experiment was conducted to investigate the performance of ZIPMS in coexistence of WiFi. The experiment was designed to ignore the effect of address assignment, thus only a small sized ZIPMS setup was required. Since the number of short addresses is limited to 65,536, as a result, the smaller the ZigBee network, the more addresses can be assigned to a ZIPMS frontend router. In this experiment, there are five NDs, one ZIPMS gateway and four frontend routers. The network tree in this experiment was set up as shown in Figure 10.

In Figure 10, the experiment network is 3-layer and the maximum number of routers is 4, i.e., Lm = 3 and Rm = 4. By using Equation (3), the Cm is obtained as 3,276, implying that each frontend router can hold 3,276 handhelds, which is much more than those needed in the experiment. Therefore, the problem of address shortage [29] has been resolved in this experiment.

In ZigBee communication, the occurrence of data loss is a very important parameter to measure the reliability of ZIPMS. Experiments were carried out to investigate the relationship of Packet Loss Probability (PLP), communication distance (d) and data rate (R) under interference of WiFi.

**Figure 11.**
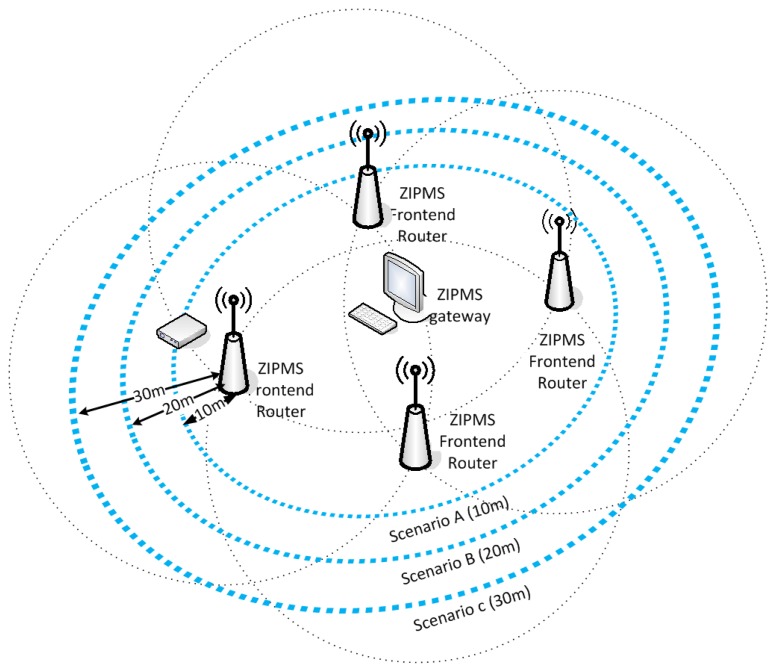
Experimental setup of reliability test.

The ZIPMS handhelds acted as biomedical sensors which send the collected medical data to the frontend routers at various date rates, from 10 kbps to 80 kbps 5,000 times. A patient carried a handheld and walked around the frontend routers at a specific distance (d) as shown in Figure 11. Three scenarios A, B, and C with d = 10, d = 20 and d = 30 respectively were investigated. For each scenario, the transmission data rate was increased from 10 kbps to 80 kbps with a step of 10 kbps.

Therefore eight cases were investigated for each single scenario. Altogether, this experiment was carried out 24 times with different values of communication distance (d) and data rate (R) and the Packet Loss Probability (PLP) was recorded at the every frontend router during the experiment. To nurture a realistic experimental environment, nine WLANs and one ZigBee were setup during the experiment. The details of frequency band usage of experimental environment are shown in Table 5.

**Figure 12.**
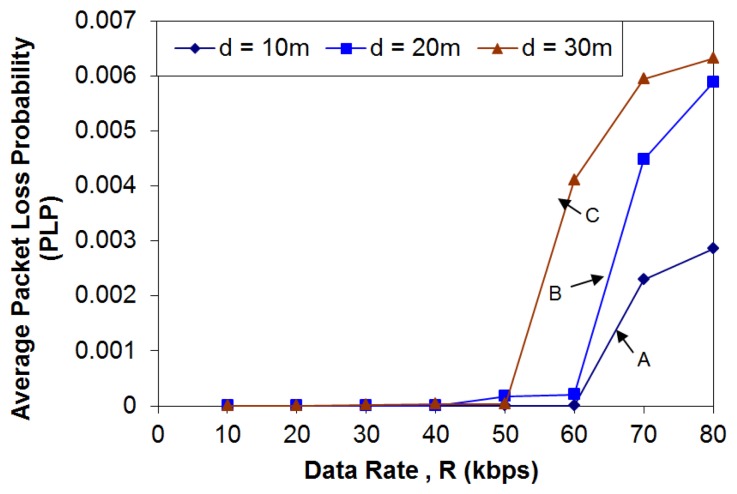
ZIPMS packet loss probability when d is 10–30 m and R is 10–80 kbps.

There were also one micro-oven and two Bluetooth handsets in the environment. Such an environment is referred to as a strong interference environment thereafter. The results obtained are shown in Figure 12.

The Packet Loss Probability (PLP) for ZIPMS under different data rate (R) and communication distance (d) conditions were shown in Figure 12. PLP is a measure of the ratio of the packet loss during the experiment for different frontend routers. It is seen that the PLP is below 0.7% for each case, irrespective of the communication distance and data rate. Despite the communication distance, PLP increases as R increases (as observed from curves A, B and C) because higher transmission data ratea may increase the chance of buffer overflow which causes data loss. Moreover, PLP of curve C is the highest, followed by curve B. Needless to say, the PLP of curve B raises the least. This is simply because of the effect of communication distance. Longer communication distance leads to weaker signal strength, therefore PLP increases as the communication distance increases. If the QoS requirement of PLP is set as 10^−4^, the maximum data rate of ZIPMS should be 40 kbps which is sufficient for all types of medical data as given in Table 1.

The achieved PLP of ZIPMS is 10^−4^, thus proving high reliability. This performance is in line with the findings from previous studies [25, 26]. ZigBee was reported to achieve 99.69% median reliability over 41 h of monitoring [25], while tree routing was reported to support 99.9%+ end-to-end delivery ratio in a clinical environment [26].

To get more insight, the impacts of the number of neighbors in the hand-off table have been studied and the duration of re-association has been recorded. In our trials of one hundred handoffs, the re-association duration ranged from 80 s to 100 s. Such a duration was only about one-third of current records [25]. An additional advantage is that handhelds always select the best LQI frontend router. Subsequently it was found that during the re-association, the number of neighbors in the hand-off table was not impacted. The encouraging results confirm that the design of ZiM2 has successfully smoothed and enabled the ZigBee hand off. With slight modifications, ZiM2 may easily be applied to other ZigBee applications.

## 7. Conclusions

In this paper, a ZigBee In-Patient Monitoring System (ZIPMS) has been proposed and developed to support telehospital applications. The system aims at providing medical data monitoring services, patient location tracking services and as well as patient paging services. Generally ZIPMS consists of a ZIPMS gateway, router frontend router and handheld. To facilitate bi-directional communications among these devices while supporting mobility, a generic mobility solution, namely ZigBee Mobile Manager (ZiM2), has been introduced. The ZiM2 is situated on top of the routing and the address assignment scheme so that it may interoperate with existing and future address assignment schemes and is also backward compatible with former ZigBee developments. The developed ZiM2 is the first of its kind in the ZigBee development. ZiM2 supports the mobility management and the address assignment from which the network capacity, the signal coverage, the data throughput, and the installation cost are investigated. Expressions describing the system planning for ZIPMS have been formulated.

For a better insight, the “Main Clinical Block and Trauma Center” of Prince of Wales Hospital is used as a show case for network planning of a ZIPMS. It is concluded that a depth of six levels will be sufficient to facilitate a ZigBee tree of about 60 nodes to support approximately 2,500 patients.

For the study of reliability testing, the packet loss probability under strong interference environment was investigated. It was found that the packet loss probability was bounded by 10^−4^ when the transmission data rate did not exceed 40 kps. Thus most, if not all, types of medical data collection can be supported. Also, the ZIPMS re-association duration ranged from 80 s to 100 s. Such performance has been improved by over 60%. With these outstanding results of QoS evaluations, ZIPMS and ZiM2 are found to perform successfully.

## Figures and Tables

**Table 1.** Medical parameters monitored by ZIPMS.

**Parameter**	**Range**	**Signal** **Frequency, Hz**	**Standard Sensor or** **Method**	**Bit Rate** **12 bits A/D**
Blood pressure, noninvasive	10–400 mmHg	60	Strain gage integrated on silicon, cuff, auscultation	1.44 kb/s
Blood pressure, invasive	10–400 mmHg	50	Strain gage integrated on silicon	1.2 kb/s
Pulmonary artery pressure (PAP)	0–50 mmHg	50	Strain gage integrated on silicon	1.2 kb/s
CVP, Central venous pressure	0–50 mmHg	50	Strain gage integrated on silicon	1.2 kb/s
ECG (3, 5 or 12 electrodes)	0.5–4 mV	250 per electrode	Skin electrodes	18 kb/s 3 electrode
Photoplethys- mography (SpO2)	80%–99%	30	Two photodiodes,	1.44 kb/s
Cardiac output	4–25 L/min	20	Thermodilution thermistor	0.48 kb/s
Body temperatures	32–40 °C	0,1	Thermistor, thermocouple	0.0024 kb/s

**Table 2.** The details of the main clinical block and trauma center.

**Detailed Items**	**Value**
Total Floor Area	75,000 m^2^
Number of Floors	13
Number of Ward/Floor	3
Number of Beds	800

**Table 3.** Typical setting of nursery ward.

**Detailed Item**	**Value**
Six Patient Ward	6
Two Patient Ward	1
Single Patient Isolation Ward	2
Total Number of Beds	40
Treatment room	1
Meeting Room	1
Rest Room	2
Bathroom	3

**Table 4.** Network configurations for the studied center.

**Lm**	**Rm**	**Cm**	**No. of ZIPMS** **Router**	**No. of Frontend** **Router**	**Supported** **ZIPMS Handheld**
6	4	48	4	52	2496

**Table 5.** Frequency band usage of testing environment.

**Channel**	**Frequency**	**Detected WLAN**	**ZigBee Network**
CH1	2.412	1	
CH2	2.417	1	
CH6	2.437	2	
CH9	2.445		1
CH9	2.452	1	
CH10	2.457	1	
CH11	2.462	3	
CH16	2.480		1 (experiment)
Total		9	2
